# The Extent of Blockade Following Axillary and Infraclavicular Approaches of Brachial Plexus Block in Uremic Patients

**DOI:** 10.4021/jocmr723w

**Published:** 2012-01-17

**Authors:** Damla Sariguney, Ahmet Mahli, Demet Coskun

**Affiliations:** a Department of Anaesthesiology and Reanimation, Gazi University Faculty of Medicine, Ankara, Turkey

## Abstract

**Introduction:**

This study was aimed to compare the axillary approach performed through multiple injection method and vertical infraclavicular approach performed through single injection method in terms of the sensory and motor block onset, quality, and extent of blocks of brachial plexus in uremic patients who underwent arteriovenous fistula surgery.

**Methods:**

Forty patients scheduled for creation of arteriovenous fistula with axillary brachial plexus block (group AX, n = 20) or infraclavicular brachial plexus block (IC group, n = 20) were examined. The median, radial, ulnar, and musculocutaneous nerves were selectively localized by nerve stimulation. The volume of the local anesthetics was calculated based on the height of each patient, and the volume determined was prepared by mixing 2% lidocaine and 0.5% bupivacaine in equal proportions. Sensory and motor block were assessed at 3, 6, 9, 12, 15, 18, and 30th min and their durations were measured.

**Results:**

While the adequate sensory and motor block rate with axillary approach was 100% in musculocutaneous, median, radial, ulnar and medial antebrachial cutaneous nerves, it was 65% in axillary nerve, 80% in intercostobrachial nerve and 95% in medial brachial cutaneous nerve. This rate was found to be 100% for all the nerves with infraclavicular approach.

**Conclusion:**

For arteriovenous fistula surgeries in uremic patients, both axillary approach performed through multiple injection method and vertical infraclavicular approach performed through single injection method can be used successfully; however, for the short performance of the procedure, infraclavicular block may be preferred.

**Keywords:**

Brachial plexus block; Axillary; Infraclavicular; Uremic patients

## Introduction

For patients with end-stage renal disease, brachial plexus block is often used to provide anesthesia for the creation or the revision of arteriovenous fistula for hemodialysis access. The use of brachial plexus block for these procedures results in analgesia and sympathetic blockade that provides optimal surgical conditions, and the adequate duration of postoperative block ensures the prevention of arterial spasm and graft thrombosis [1]. The use of both axillary and infraclavicular approach for brachial plexus block is indicated for hand and arm surgery [2].

Previosuly, Niemi et al [3] compared axillary approach and infraclavicular coracoid approach performed through single injection method for arteriovenous fistula surgeries in uremic patients and at the end of their study, both approaches were reported to have similar efficacies. In this study, we aimed to compare the axillary approach performed through multiple injection method and vertical infraclavicular approach performed through single injection method in terms of sensory and motor block onset, quality, and extent of blocks of brachial plexus by the use of some local anesthetics volume containing a mixture of lidocaine and bupivacaine in equal amounts for arteriovenous fistula surgery in uremic patients.

## Methods

This study was approved by the Institutional Ethics Committee, and informed consent was obtained from each patient. The patient population included patients of American Society Anesthesiologists (ASA) physical status III, ages 19 - 80 years, scheduled for creation or revision of arteriovenous fistula of distal upper extremity. Patients were excluded if they had a history of neurological, neuromuscular, or psychiatric disorders or hepatic, respiratory, or cardiac disease. Patients with a history of drug or alcohol abuse, coagulation disorders, uncontrolled seizures, and pregnant or lactating women were excluded as well.

Patients were randomized in two groups of 20 to receive either axillary brachial plexus block (group AX, n = 20) or infraclavicular brachial plexus block (IC group, n = 20). Randomization was based on an investigator-generated code that was sealed in sequentially numbered opaque envelops.

No premedication was given to the patients, since full cooperation during block assessment was required. All the patients included in the study were chronic haemodialysis patients and they had received haemodialysis treatment one day before the block performance. Their routine laboratory examinations were made preoperatively. Prior to the procedure, all patients had normal prothrombin (PT) and partial thromboplastin (PTT) times. On arrival in the anesthetic room, an intravenous catheter was placed in the upper limb contralateral to the surgical site and saline solution was given at a rate of 2 mL/kg/hour. Monitoring included electrocardiography, non-invasive blood pressure and pulse oximetry. Supplemental oxygen (via nasal cannula at 4 L/minute) was applied throughout the procedure. The blocks in two groups were performed via peripheral nerve stimulator (Stimuplex HNS® 11; B. Braun, Melsungen, Germany) and short-beveled stimulating needle (Stimuplex® Kanule A, 50 mm; B. Braun, Melsungen, Germany).

The perivascular axillary approach was performed in a supine patient with the upper arm abducted 90º, and flexed 90º cranially at the elbow with a supinated forearm. After identification of the axillary artery, the needle was inserted as high as possible in the axilla superior and tangential to the axillary artery [4]. The vertical infraclavicular approach was also performed on the supine patient with the upper arm along the side, but with the elbow flexed and the hand resting on the lower chest or abdomen. After identification of the landmarks, the puncture site was marked half way between the jugular notch and the most ventral part of the acromion [5]. The time taken for the block procedure included the positioning of the patient, indicating the landmarks and performing the actual block.

For both approaches, the volume of the local anesthetics (approximately 30 - 35 mL) was calculated based on the height of each patient according to the formula ”volume (mL) = height (cm)/5“ [6], and the volume determined was prepared by mixing 2% lidocaine and 0.5% bupivacaine in equal proportions. In all the patients undergoing the procedure, the plexus was identified with a short-beveled electric stimulation needle connected to a nerve stimulator by using a low current (< 1.0 mA).

For axillary approach, the median, radial, ulnar, and musculocutaneous nerves were selectively localized by elicited characteristic muscle group movements secondary to each nerve stimulation. After obtaining an appropriate peripheral motor response with a current near or below 0.5 mA with respect to the stimulation of each nerve, predetermined volumes of local anesthetics in accordance with the formula was selectively injected onto each nerve through multiple injections in the AX group, with intermittent aspiration. Firm digital pressure was maintained during the injection and 3 minutes thereafter immediately distal to the injection site to prevent distal flow of the local anesthetic solution. The arm was then brought to rest at the patient’s side.

For infraclavicular approach, the current was reduced until appropriate motor response in hand (finger movements) or wrist (flexion or extension) was achieved near or below 0.5 mA and then predetermined volumes of local anesthetic in accordance with the formula was injected over one minute, with repeat aspirations every 5 mL. Verbal contact with the patients was maintained throughout the injection, and before the injections were made, the patients were informed about the signs of local anesthetic toxicity, such as numbness of the lips and tongue, and lightheadedness.

Sensory and motor blocks of all the upper extremity nerves were evaluated at the 3rd, 6th, 9th, 12th, 15th, 18th, and 30th minute after injection and recorded on a chart. The patients were followed up for 24 hours including both the intraoperative and the postoperative periods. During that period, the side effects and complications were recorded. Sensory block was assessed in the area propria of the sensory nerves by pinprick using the blunt end of a 27-gauge dental needle and was graded according to the following the rating scale [7]: 0 = sharp, 1 = dull (analgesia), and 2 = no sensation (anesthesia). Motor block was tested using six different nerves. The motor block quality was evaluated based on the function of the muscle innervated by each nerve by observing the motion of the related muscle in each patient and the degree of the motion. The rating scale [7] for motor block was: 0 = normal contraction, 1 = reduced contraction (paresis), and 2 = no contraction (paralysis). The frequencies of sensory and motor block of different nerves of the upper extremity were determined for each of the two approaches. For clarity, either analgesia or anesthesia was evaluated as indicative of adequate sensory block. Additionally, either paresis or paralysis was evaluated as indicative of the adequate motor block. Before the operation, a pinprick test was conducted in the operation site, and if pain was felt (inadequate sensory block), additional peripheral nerve block was provided by the injection of 3 - 5 mL of 2% lidocaine. Requirements for additional local anesthetic infiltration and the incidence of complications were noted. After the operation, patients were monitored in the postanesthesia care unit (PACU) and were discharged from the hospital when recovery from sensory and motor blockade occured.

SPSS version 14 statistical software (SPSS, Chicago, III) was used to perform statistical analysis. The results are expressed as mean values with standard deviation. An unpaired Student t-test or a Mann-Whitney U test was used to compare the demographic variables and operative data. For the analysis of the quality of the block, a chi-square or Fisher exact test was used. Differences were regarded as statistically significant if P < 0.05.

## Results

Demographic data and duration of operation were not significantly different between the groups. However, statistically significant differences were observed (P = 0.0001) in terms of the durations of block procedure (Table 1).

**Table 1:** Patients’ Characteristics and Duration of Operation and Block Procedure (mean ± SD)

**Groups**	**AX (n = 20)**	**IC (n = 20)**	**P value**
Gender (M/F)	14/6	10/10	NS
Age (year)	55.1 ± 19.8	51.0 ± 15.5	NS
Weight (kg)	63.4 ± 13.7	64.1 ± 12.8	NS
Height (cm)	167.6 ± 9.5	162.4 ± 9.6	NS
Duration of operation (minute)	77.1 ± 21.7	81.5 ± 18.6	NS
Duration of block procedure (minute)	13.7 ± 4.0	4.23 ± 2.4	0.0001

AX: Axillary; IC: Infraclavicular; NS: Not Significant.

No statistically significant differences were found between the two groups in terms of the preoperative laboratory values (Table 2).

**Table 2:** Preoperative Laboratory Values (mean ± SD)

**Venous blood gas and electrolytes**	**AX (n=20)**	**IC (n=20)**	**P value**
BUN (mg/dL)	51.7 ± 17.8	47 ± 15.9	NS
Creatinine (mg/dL)	5.3 ± 1.8	4.1 ± 1.7	NS
Hemglobine (gr/dL)	9.1 ± 1.3	9.8 ± 1.4	NS
K (mEq/L)	4.2 ± 0.8	4.2 ± 0.6	NS
Ca (mg/dL)	7.9 ± 0.7	8.6 ± 0.8	NS
			

AX: Axillary, IC: Infraclavicular; NS: Not Significant.

Since axillary and infraclavicular approaches were used in this study, the evaluation of the sensory and motor nerves starting from the onset of the block until the 30th minute revealed that the block rate of each nerve was slower or faster than or parallel to each other. In order to allow the onset of surgery and provide anesthesia throughout the operation, the quality of the sensory and motor block at the 30th minute is important for us to be able to determine whether an additional peripheral block is needed. None of the patients in any of the groups required additional peripheral nerve block.

When the axillary nerve was assessed, statistically significant differences were observed between the groups at all assessment times, that is, at the 3rd,6th, 9th, 12th, 15th, 18th, and 30th minutes in terms of sensory block (Table 3) and at the 9th, 12th, 15th, 18th, and 30th minutes in terms of motor block (Table 4). At the 30th minute the ratio of adequate sensory and motor blocks for axillary nerve in groups AX and IC were 65% and 100%, respectively.

**Table 3:** Development of Sensory Block With Axillary (AX) and Infraclavicular (IC) Approach of Brachial Plexus Block

**Nerves**	**3 min**	**6 min**	**9 min**	**12 min**	**15 min**	**18 min**	**30 min**
Axillary							
AX	19/1/0	18/2/0	16/4/0	14/6/0	12/8/0	10/10/0	7/13/0
IC	13/7/0*	11/9/0*	8/12/0*	2/15/3*	2/20/3*	1/11/8*	0/10/10*
Musculocutaneous							
AX	10/10/0	6/12/2	4/11/5	1/11/8	0/9/11	0/8/12	0/6/14
IC	12/8/0	4/16/0	0/16/4	0/12/8	0/4/16	0/3/17	0/2/18
Radial							
AX	9/10/1	3/16/1	1/16/3	0/13/7	0/11/9	0/11/9	0/10/10
IC	13/7/0	8/12/0	2/15/3	0/14/6	0/8/12	0/6/14	0/3/17
Median							
AX	9/10/1	5/14/1	3/13/4	2/9/9	1/9/10	1/8/11	0/6/14
IC	13/7/0	5/15/0	0/18/2	0/14/6	0/7/13	0/3/17	0/1/19
Ulnar							
AX	5/14/1	2/12/6	1/9/10	0/7/13	0/5/15	0/4/16	0/3/17
IC	16/3/1*	11/8/1*	4/14/2*	1/16/3*	1/12/7*	1/11/8*	0/8/12*
Medial Antebr Cut							
AX	5/14/1	4/10/6	2/9/9	1/6/13	0/6/14	0/4/16	0/1/19
IC	16/4/0*	12/8/0*	6/13/1*	3/15/2*	1/11/8	0/8/12	0/4/16
Medial Brach Cut							
AX	10/10/0	9/11/0	7/13/0	7/12/1	6/11/3	4/13/3	1/15/4
IC	14/6/0	11/9/0	6/14/0	3/16/1	0/13/7*	0/10/10*	0/7/13*
Intercostobrachial							
AX	10/9/1	10/9/1	8/9/3	6/11/3	5/12/3	5/12/3	4/13/3
IC	17/3/0	12/8/0	5/14/1	2/13/5	1/12/7	1/9/10*	0/7/13*

Number of patients with ”sharp/dull/no sensation“ to pinprick are shown. P < 0.05 (AX versus IC).

**Table 4:** Development of Motor Block With Axillary (AX) and Infraclavicular (IC) Approach of Brachial Plexus Block

**Nerves**	**3 min**	**6 min**	**9 min**	**12 min**	**15 min**	**18 min**	**30 min**
Axillary							
AX	17/3/0	16/4/0	12/8/0	12/8/0	9/11/0	9/11/0	7/13/0
IC	11/8/1	9/10/1	4/12/4*	1/14/5*	0/12/8*	0/10/10*	0/8/12*
Musculocutaneous							
AX	6/12/2	4/11/5	1/12/7	0/10/10	0/6/14	0/6/14	0/5/15
IC	8/12/0	4/12/4	0/11/9	0/6/14	0/3/17	0/2/18	0/1/19
Radial							
AX	7/10/3	4/11/5	0/10/10	0/8/12	0/6/14	0/3/17	0/2/18
IC	11/8/1	4/14/2	0/15/5	0/8/12	0/4/16	0/1/19	0/0/20
Median							
AX	7/13/0	5/13/2	3/7/10	0/10/10	0/8/12	0/8/12	0/7/13
IC	13/7/0	4/14/2	0/14/6	0/13/7	0/4/16	0/3/17	0/1/19
Ulnar							
AX	5/11/4	1/8/11	1/7/12	0/4/16	0/4/16	0/2/18	0/0/20
IC	16/3/1*	10/9/1*	5/14/1*	2/14/4*	0/12/8*	0/10/10*	0/7/13*

Number of patients with motor power as ”normal contraction/reduced contraction/no contraction“ of the hand and arm are shown. P < 0.05 (AX versus IC).

When musculocutaneous, radial, and median nerves are assessed in terms of both sensory and motor block, no statistically significant differences were observed between the AX group and IC group at all assessment times. At the 30th minute, the ratio of adequate sensory and motor blocks for musculocutaneous, radial, and median nerves for both group AX and group IC were found to be 100% (Table 3 and 4).

When the ulnar nerve was assessed, statistically significant differences were observed in terms of both sensory and motor blocks at all assessment times, that is, at the 3rd,6th, 9th, 12th, 15th, 18th, and 30th minutes (Table 3 and 4). At the 30th minute, the ratios of adequate sensory and motor blocks for ulnar nerve in groups AX and IC were found to be 100%.

Statistically significant differences were observed between AX and IC groups in terms of sensory block (Table 3) at the 3rd, 6th, 9th, and 12th minutes, in medial antebrachial cutaneous at the 15th, 18th, and 30th minute in medial brachial cutaneous nerves, and at the 18th and 30th minutes in intercostobrachial nerve. At the 30th minute, the adequate sensory block ratios in medial antebrachial cutaneous were found to be 100% in both groups. The adequate sensory block ratios in medial brachial cutaneous and intercostobrachial nerves were found to be 100% in the IC group, and 95% and 80% in the AX group, respectively.

The motor and sensory block speed of axillary nerve, and the sensory block speed of medial brachial cutaneous and intercostobrachial nerves were found to be higher in the IC group than in the AX group. However, the sensory and motor block speed of ulnar nerve, and the sensory block speed of medial antebrachial cutaneous was found to be higher in group AX than in group IC, and the difference was statistically significant.

No statistically significant differences were found between the two groups in terms of the mean arterial pressure values at all measurement times and they were within the nomal limits. Signs of toxicity related to local anesthetics were not noted in any of the patients in any of the groups.

## Discussion

The block of brachial plexus with axillary approach is frequently preferred in a variety of orthopedic and soft tissue surgical procedures of the upper extremity, as well as for patients with end-stage renal disease for whom arteriovenous fistula creation or revision is required for hemodialysis access [8]. For the creation of an arteriovenous fistula for hemodialysis, succesful musculocutaneous nerve block is usually essential in order to provide adequate anesthesia as this nerve innervates the lateral aspect of the distal forearm. In cases where the exploration of blood vessels is extended distally, analgesia of the median and the radial nerve may also be required [3].

Axillary approach is relatively safe and, if dosage limits are observed, complications are uncommon [9]. Multiple injection techniques using nerve stimulation for axillary plexus block provide more effective anesthesia when compared to single injection techniques [10]. Infraclavicular block is a safe and simple technique to provide surgical anesthesia for the lower arm, and the resulting efficacy is comparable to other brachial plexus blocks. Besides, when compared to a single-injection axillary block, it provides a more reliable blockade of the musculocutaneous and axillary nerves [2,11]. However, it has been reported that infraclavicular block and a multiple-stimulation axillary block provide comparable efficacy [12].

In a study where Koschielniak-Nielsen et al [13] compared infraclavicular approach to axillary approach performed through multiple injection method, it was reported that patients on whom infraclavicular approach was used had poorer analgesia of the ulnar and the medial cutaneous brachial nerves after the initial block, but marginally better analgesia of the axillary nerve. In another study where Kapral et al [14] compared infraclavicular approach to axillary approach performed through single injection method, it was reported that motor block of the axillary and musculocutaneous nerves as well as sensory block of the axillary, median brachial cutaneous and musculocutaneous nerves had a significantly superior block in patients on whom infraclavicular approach was used when compared to those on whom axillary approach was used.

In our study, as well as in the studies mentioned above, in patients on whom brachial plexus was performed through infraclavicular approach, sensory and motor blocks of axillary, musculocutaneous, and medial brachial cutaneous nerves in addition to median, radial, and intercostobrachial nerves were found to be superior to the outcomes of the axillary approach. On the other hand, in patients on whom axillary approach was used, the sensory and motor block of the ulnar and medial antebrachial cutaneous nerves were found to be superior to the outcomes of the other approach.

Even though the performance of axillary block through multiple injection method provides the successful block of the musculocutaneus nerve [12], which is also indicated by the 100% adequate block rate of the musculocutaneus nerve in our study, 65% adequate block rate of axillary nerve and 80% adequate block rate of intercostobrachial nerve was obtained in spite of the axillary approach through multiple injection method. The risk of spreading the local anesthetics to the distal, which is accepted as the primary failure reason in axillary block, is directly eliminated in infraclavicular block, and this is one advantage of infraclavicular block to axillary block [13]. This also explains the superiority of motor and sensory block of axillary and musculocutaneous nerves in the infraclavicular group. These results are consistent with those of the studies conducted by Kilka et al [5] and Whiffler [15]. However, in a study where Coskun and Mahli [16] compared axillary approach performed through multiple injection method to supraclavicular and interscalen approaches, excluding intercostobrachial nerve, where the adequate sensory was found to be 48%, the adequate sensory and motor block ratio in the axillary group on the nerves of brachial plexus was found to be 100% and 92 - 100%, respectively.

In one study, Niemi et al [3] compared axillary approach and infraclavicular coracoid approach performed through single injection method for arteriovenous fistula surgeries in uremic patients. They reported that blockade of the musculocutaneous nerve developed faster with the infraclavicular coracoid approach than with the other and the infraclavicular coracoid approach may be preferable in patients scheduled for creation of an arteriovenous fistula at the forearm. In the same study, the researchers observed surgical analgesia in 90 - 97% of the test territories of the ulnar, median radial, and musculocutaneous nerves with the infraclavicular coracoid approach.

In our study, the following adequate motor and sensory block rates were observed in axillary approach: 65% in axillary nerve, 80% in intercostobrachial nerve, 95% in medial brachial cutaneous nerve, and 100% in musculocutaneous, median, radial, ulnar and medial antebrachial cutaneous nerves. The adequate motor and sensory block rate in infraclavicular approach is 100% in all nerves.

The average duration of procedure, which was found to be 4.2 minutes in patients on whom infraclavicular approach was performed, is similar to 3.35 minutes found in the study conducted by Maria and Tielens [17]. The average duration of procedure in patients on whom axillary approach was performed was found to be 13.67 minutes. In the study where Koschielniak-Nielsen et al [18] compared infraclavicular approach to axillary approach performed through multiple injection technique, the durations of the procedures were found to be significantly shorter in infraclavicular approach. Similarly, when the durations of procedures found in our study were compared, it was observed that the duration was significantly shorter in patients on whom infraclavicular approach was performed.

In this study, we aimed to compare the axillary approach performed through multiple injection method and vertical infraclavicular approach performed through single injection method in terms of sensory and motor block onset, quality, and extend of blocks of brachial plexus by the use of some local anesthetics volume containing a mixture of lidocaine and bupivacaine in equal amounts for arteriovenous fistula surgery in uremic patients. While with infraclavicular approach the adequate block rate was found to be 100% in all nerves, with axillary approach, 100% adequate block rate could not be observed in axillary, intercostobrachial and medial brachial cutaneous nerves. However, additional block was not required for any patient on whom arteriovenous fistula surgery was performed according to the innervation state of the surgical site.

### Conclusion

Both axillary approach performed through multiple injection method and vertical infraclavicular approach performed through single injection method can be used successfully for arteriovenous fistula surgeries in uremic patients; however, infraclavicular block may be preferred for the short performance of the procedure.
